# Increasing diagnostic effectiveness of thyroid nodule evaluation by implementation of cell block preparation in routine US-FNA analysis

**DOI:** 10.1590/2359-3997000000180

**Published:** 2016-08-01

**Authors:** Ana Patrícia de Cristo, Heloísa Folgierini Goldstein, Carlo Sasso Faccin, Ana Luiza Maia, Marcia Silveira Graudenz

**Affiliations:** 1 Universidade Federal do Rio Grande do Sul Hospital de Clínicas de Porto Alegre Porto Alegre RS Brasil Programa de Pós-Graduação em Medicina: Endocrinologia, Universidade Federal do Rio Grande do Sul (UFRGS); Hospital de Clínicas de Porto Alegre (HCPA), Porto Alegre, RS, Brasil; 2 Hospital de Clínicas de Porto Alegre Universidade Federal do Rio Grande do Sul Porto Alegre RS Brasil Serviço de Patologia, Hospital de Clínicas de Porto Alegre (HCPA); Universidade Federal do Rio Grande do Sul (UFRGS), Porto Alegre, RS, Brasil; 3 Hospital de Clínicas de Porto Alegre Porto Alegre RS Brasil Serviço de Radiologia, Hospital de Clínicas de Porto Alegre (HCPA), Porto Alegre, RS, Brasil; 4 Hospital de Clínicas de Porto Alegre Universidade Federal do Rio Grande do Sul Porto Alegre RS Brasil Unidade de Tireoide, Serviço de Endocrinologia, Hospital de Clínicas de Porto Alegre (HCPA), Universidade Federal do Rio Grande do Sul (UFRGS), Porto Alegre, RS, Brasil

**Keywords:** Ultrasound-guided fine-needle aspiration (US-FNA), cell block, thyroid nodule, thyroid cancer

## Abstract

**Objective:**

Ultrasound-guided fine-needle aspiration (US-FNA) biopsy has proven to be an accurate and efficient tool in thyroid nodule evaluation. We evaluated whether cell block adds to the diagnostic accuracy of US-FNA.

**Subjects and methods:**

Three hundred twenty-eight consecutive patients underwent US-FNA, cytology and cell block evaluation. Six slides were prepared for each patient and stained by Papanicolaou and Giemsa techniques. The residual hemorrhagic aspirate in the syringe and needle was fixed in 10% formalin and paraffin-embedded (cell block). The histological sections were examined as a complementary diagnostic tool to US-FNA.

**Results:**

The study population comprised 89% females and the mean age was 57.4 ± 13.7 years. The mean nodule size was 2.3 ± 1.2 cm. US-FNA cytological results were as follows: Bethesda I, 17.1% (n = 56); Bethesda II, 61.6% (n = 202); Bethesda III, 9.5% (n = 31); Bethesda IV, 5.8% (n = 19); Bethesda V, 2.4% (n = 8), and Bethesda VI, 3.6% (n = 12). Cell blocks were obtained in 100% of cases and were considered diagnostic in 89.6%. Combined cytological and cell block (cyto-cell block) results were as follows: unsatisfactory, 4.3% (n = 14); benign, 72.6% (n = 238); indeterminate, 11.3% (n = 37); follicular lesion, 5.8% (n = 19); suspicious for malignancy, 2.4% (n = 8), and malignant, 3.6% (n = 12). The sensitivity and specificity for cyto-cell block was 100% and 90%, respectively, and the accuracy was 94%. Cyto-cell block analysis reduced the rate of unsatisfactory samples (p < 0.001).

**Conclusions:**

The cyto-cell block interpretation improved the efficiency of US-FNA. This simple, fast and low-cost technique should be used as an adjunctive test in thyroid nodule evaluation. Arch Endocrinol Metab. 2016;60(4):367-73

## INTRODUCTION

Thyroid nodules are common clinical findings, more frequent in women and in the elderly population, while cases of thyroid cancer are relatively rare. The current diagnostic method of choice for differentiating benign from malignant lesions is the fine needle aspiration (FNA) cytology guided by ultrasonography (US-FNA) (1-3). This procedure has been shown to be superior to clinical, radionucleotide or thyroid ultrasound assessment alone (4). Technique limitations and its advantages are well established and described by several authors (1,5). According to the current guidelines of the Brazilian Endocrine Society (2013) and the American Thyroid Association (2009), when hyperfunctioning or purely cystic nodules have been ruled out, US-FNA is a fundamental tool on thyroid nodule evaluation (6).

Thyroid carcinoma is the most common endocrine tumor of the head and neck region, accounting for around 5% all cancers in women. The latest global estimate indicated the occurrence of about 300.000 new cases of this cancer (68.000 in men and 230.000 in women). In Brazil, it corresponds to 1.150 new cases of thyroid cancer in males and 8.050 in females, with an estimated risk of 1.15/100.000 cases in men and 7.91/100.000 cases in women, according to data recorded by INCA (National Cancer Institute) in the year 2014 (7,8).

In the last years, there has been significant progress in the adoption of thyroid US-FNA reporting terminology due to international efforts. From the deliberations in October 2007 by the National Cancer Institute in Bethesda, Maryland, the diagnostic categories for thyroid US-FNA were defined following the “Bethesda System”. The Bethesda System, consists of 6 categories and each has distinct morphological characteristics and cancer risk. These guidelines facilitate communication between professionals and help in the research and diagnosis of thyroid disease. Thus, the system allows easy data sharing between different laboratories nationally and internationally (9). Nevertheless, wide variations are reported in the literature in the overall performance of thyroid US-FNA as a diagnostic test, which reflect either variations in methodology used or variations in criteria for reporting of thyroid US-FNA (10).

The US-FNA technique has some inherent limitations. First, the US-FNA sample must be evaluated for adequacy. Inadequate samples are reported as “nondiagnostic” (ND) or “unsatisfactory” (UNS). This category applies to specimens that are unsatisfactory owing to obscuring blood, overly thick smears, air drying of alcohol-fixed smears, or an inadequate number of follicular cells. ND/UNS results occur in 2% to 20% of cases but ideally should be limited to no more than 10% of thyroid US-FNAs. Furthermore, some thyroids US-FNAs are not easily classified into the benign, suspicious, or malignant categories. Such cases represent a minority of thyroid US-FNAs and in the Bethesda System are reported as “atypia of undetermined significance” (AUS) or “follicular lesion of undetermined significance”. An AUS result is obtained in 3% to 6% of thyroid US-FNAs (9).

Cell blocks are a valuable tool to evaluate various cytology specimens and are often prepared with US-FNA from multiple organs as an adjunct to smears in the diagnosis of aspirated lesions. In addition to the architectural details of the specimen, cell blocks allow for evaluation by ancillary studies such as immunohistochemistry. The cell block technique includes the centrifugation of the material aspirated from the lesion to decrease cell dispersion of traditional cytology smears, which presence of hemorrhage or low cellularity sometimes makes the method unsatisfactory (10-12).

The aim of this study was to evaluate the diagnostic efficiency of US-FNA followed by processing the aspirated material using the cell block technique as part of investigation of thyroid nodules by a multidisciplinary team in a tertiary, University-based Hospital.

## SUBJECTS AND METHODS

### Patients

Over a period of 22 months (March 2012 to December 2013), 559 patients were attended at the Thyroid Unit of the Centro de Pronto Diagnóstico Ambulatorial (CPDA), Hospital de Clínicas de Porto Alegre (HCPA). Two-hundred-one (36%) patients were excluded from analysis: 155 (27.7) did not meet criteria for FNA-US and 46 had aspirated cervical lymph nodes. A total of 358 (64%) patients presented with thyroid nodules underwent US-FNA procedure were initially included in the study. Out of them, 328 (91.6%) patients had their samples analyzed by both cyto and cyto-cell while 30 samples (8.3%) were analyzed only by cytology. Thus, the study population consisted of 328 consecutive patients.

### US-FNA, cyto-cell block

All patients underwent thyroid US-FNA performed by the same radiologist using a high-resolution ALOKA ultrasound device with a 7.5 MHz linear transducer (Tokyo, Japan). US-FNA was performed with a disposable needle (21 G) connected to a 10 ml disposable syringe. The needle tip was identified as a bright spot in the monitor and then inserted perpendicularly to the cervical region and guided to the aspiration site. Constant negative pressure with the syringe plunger is maintained until material is seen at the base of the needle. Multidirectional aspiration was performed in: dominant nodules in patients with multinodular goiter, suspicious nodules on ultrasonography or in various locations in cases of diffuse goiter. Rapid on-site evaluation of fine needle aspiration of all specimens were performed to evaluate adequacy. For a thyroid FNA specimen to be considered satisfactory, at least 6 groups of follicular cells were required, each group composed of at least 10 cells (9). Cyst-fluid-only (CFO) cases were considered ND/UNS. Immediate on site reaspiration was performed in cases considered inadequate for diagnosis. Six cytology slides were prepared for each patient, four of them air dried and immediately stained by May Grünwald Giemsa technique. The other two slides were immediately fixed in ethanol 96º and subsequently stained by the Papanicolaou technique. The residual hemorrhagic aspirate in the syringe and needle was fixed in 10% formalin, transferred to a test tube and centrifuged at 600 rpm for 30 minutes. The pellet obtained after centrifugation was transferred to absorbent paper, placed in a histological cassette and fixed in formaldehyde solution 10% for 24 hours. After the material was processed routinely (automatic processor), following the dehydration steps (ethanol), diaphanization (xylene), impregnation and paraffin embedding, microtome and HE staining technique. Histological sections stained with Hematoxylin and Eosin were examined as a complementary diagnostic tool to US-FNA.

Two independent pathologists reviewed the cytological and histological slides of each case together and a final diagnostic category was assigned to cyto and cyto-cell block. The adequacy of cytological aspirates was defined according to the recommendations of the *Papanicolaou Society of Cytopathology Task Force on Standards of Practice*, 1996 (13) and cytological results classified according to the criteria of the Bethesda System for Cytological Classification of Thyroid Nodules into 6 diagnostic categories: 1) Non-diagnosis or Unsatisfactory, 2) Benign, 3) Atypia of undetermined significance; 4) Follicular neoplasm or suspicious for follicular neoplasm; 5) Suspicious for malignancy and 6) Malignant.

Cell-block slides were reviewed for the presence of cellular elements and classified into two categories: 1) diagnostic and 2) non diagnostic.

US-FNA cytology and cell block results were validated in a subgroup of 75 patients who underwent lobectomy or thyroidectomy at our hospital and the surgical specimens examined at the Pathology Laboratory. The cytological and cyto-cell block results were classified as 1) false-negative for malignancy. 2) false-positive for malignancy, 3) true positive and 4) true negative. True-negatives were Bethesda Class II cytology cases with benign pathology reports. True positives were suspicious for malignancy (Bethesda Class V) and malignant (Bethesda Class VI) confirmed malignant by the final pathology report.

Statistical analysis of the results was performed with SPSS software (Statistical Package for Social Sciences) version 18.0 and based on frequency data and mean ± standard deviation (SD). To evaluate the diagnostic accuracy of US-FNA, the rates of sensitivity, specificity, negative predictive value and positive predictive value were calculated (14). P value was considered statistically significant for values less than 5% (p < 0.05).

The project was approved by the Research Ethics Committee of the Hospital de Clínicas de Porto Alegre (GPPG 140538).

## RESULTS

### Patients

A total of 358 patients with thyroid nodules underwent US-FNA procedure. Of them, 328 (91.6%) had their samples analyzed by both cyto and cyto-cell while 30 samples (8.3%) were analyzed only by cytology. Thus the study population consisted of 328 consecutive patients (291 females, 88.7%). Mean age was 57.4 ± 13.7 years and the mean size of the nodules was 2.31 ± 1.22 cm. No significant differences were observed on gender (90% females), mean age (50.3 ± 14.8 years) or nodular size (2.09 ± 0.87 cm) between patients who had cell block and those who not (all P > 0.10).

### Cytology and cell block

The cytological results were as follows: 17.1% (n = 56) classified as Bethesda I; 61.6% (n = 202), Bethesda II; 9.5% (n = 31), Bethesda III; 5.8% (n = 19), Bethesda IV; 2.4% (n = 8), Bethesda V and 3.6% (n = 12) Bethesda VI.

**Figure 1:**
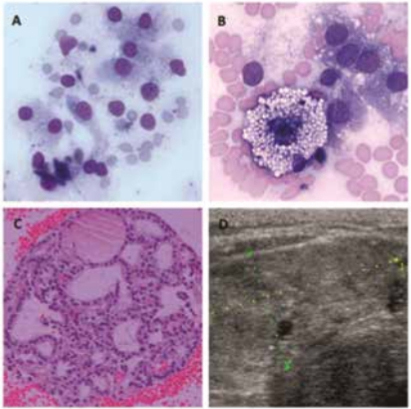
Benign follicular nodule (Bethesda II). A and B: Typical follicular cells containing green-black cytoplasmic granules and vacuolated macrophage (smear, May Grünwald-Giemsa, 400X). C: Follicular fragment showing hyperplastic architecture (cell block, H&E stain, 200X). D: US image of an isoechoic benign nodule with regular margins and no microcalcifications.

**Figure 2:**
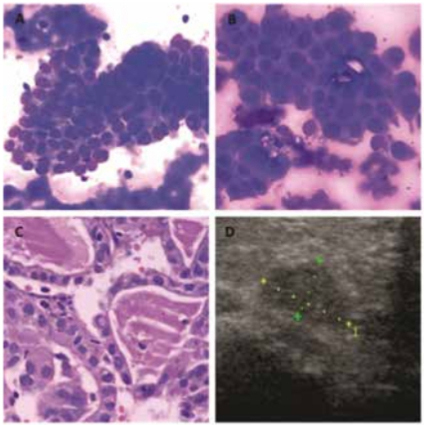
Papillary Thyroid Carcinoma (Bethesda VI). A and B: Monolayer sheets of atypical cells with a syncytial-like appearance and dense cytoplasm. Nuclear grooves are not conspicuous in air-dried preparations (smear, May Grünwald-Giemsa, 400X). C: Follicular fragments with nuclear pseudoinclusions (cell block, H&E stain, 400X). D: US image showing an irregular hypoechoic nodule with microcalcifications.

Cell blocks were obtained in 328 cases and were considered diagnostic in 89.6% and not diagnostic (unsatisfactory) in 34 cases (10.4%). The combined cytological and cell block (cyto-cell) interpretations were as follows: 4.3% (n = 14) unsatisfactory, 72.6% (n = 238) benign, 11.3% (n = 37) indeterminate, 5.8% (n = 19) follicular lesion, 2.4% (n = 8) suspicious for malignancy and 3.6% (n = 12) malignant (Table 1). The comparison of unsatisfactory ratios between the two techniques, US-FNA cytology and cyto-cell block, showed a significant reduction in the unsatisfactory rate (17.1% vs. 4.3%, p < 0.001). Illustrative cases of combined cytological, cell block and US results classified as benign and malignant are observed in Figures 1 and 2, respectively.

**Table 1:** Distribution of the diagnostic categories in 328 patients and 75 cases operated

Diagnoses	Cytology N (%)	Cyto-cell block N (%)	Final histology
Unsatisfactory (non diagnostic)	56 (17.1)	14 (4.3)	3 MNG
Benign follicular nodule/thyroiditis	202 (61.6)	238 (72.6)	17 MNG, 1 thyroiditis, 1 follicular adenoma, 1 nodular hyperplasia and 1 PTMC
Atypical features	31 (9.5)	37 (11.3)	9 MNG, 1 thyroiditis, 2 follicular adenomas, 1 nodular hyperplasias, 1 Hürthle cell adenoma, 1 PTC and 1 PTMC
Follicular lesions	19 (5.8)	19 (5.8)	7 MNG, 5 follicular adenomas, 1 nodular hyperplasia, 3 Hürthle cell carcinomas and 1 PTMC
Suspicious for malignancy	8 (2.4)	8 (2.4)	2 MNG and 7 PTCs
Malignant	12 (3.6)	12 (3.6)	1 MTC and 8 PTCs
Total	328	328	75

MNG: multinodular colloid goiters; PTC: papillary thyroid carcinoma; PTMC: papillary thyroid microcarcinoma; MTC: medullary thyroid carcinoma.

### Cyto-cell block and histological follow-up

Seventy-five out of 328 (22.8%) patients underwent surgery and the anatomopathology report was considered as the gold standard diagnosis. All patients had cytological and cell block reports. The results are shown in Table 1. Three patients included in category I (unsatisfactory) were diagnosed as multinodular goiter (MNG). Twenty-one patients classified as class II had benign findings in the surgical pathology report (17 MNG, 1 thyroiditis, 1 follicular adenoma, 1 nodular hyperplasia); papillary thyroid microcarcinoma (PTMC) was found incidentally in 1 case. The patients classified as class V had 2 MNG and 7 papillary thyroid carcinoma (PTC) whereas patients classified in category VI had 1 medullary thyroid carcinoma (MTC) and 8 PTCs.

Overall, the number of cases that had their final diagnosis changed after cell block analysis was 44/328 (13.4%). The majority of cases belonged to the Bethesda I category (40/44 or 91%) and was changed to a Bethesda II, or less frequently, to a Bethesda III category. The remaining cases (3/44 or 6.8%) were Bethesda II that changed to Bethesda III or the reverse. Just one Bethesda II case in cytology has become Bethesda VI after the analysis of the cell block (Table 2). The surgery in this case was indicated by the attending physician after the cell block analysis showing papillary fragments with nuclear atypia and pseudoinclusions, suggesting papillary thyroid carcinoma. Subsequently, the anatopathogical examination revealed papillary carcinoma.

**Table 2:** Samples with diagnosis changed after cell block analysis

Number of cases (N = 44) N (%)	Cytology (Bethesda System)	Cyto-cell block
36 (81%)	I	Benign
4 (9%)	I	Atypical Features
2 (4.5%)	II	Atypical Features
1 (2.2%)	III	Benign
1 (2.2%)	II	Malignant

### Cytology/Cyto-cell block Accuracy

Cytology analysis of sensitivity and specificity were calculated considering 35 cases for analysis. The calculated sensitivity and specificity on cytology was 93% (15/16) and 89% (17/19), respectively. The analysis of positive predictive value and negative predictive value revealed, respectively, 88% and 94%. The accuracy was 91% (32/35) (Table 3). The results included 2 false-positives for malignancy, 1 false-negative, 15 truly positive and 17 truly negative cases. Cases excluded from the analysis of sensitivity and specificity were as follows: 9 unsatisfactory cases (class I), 13 indeterminate cases (class III), 16 follicular lesions (class IV) and 2 PTMCs detected incidentally. PTMC was classified as ‘‘incidental’’ when it was found at histological examination after thyroid surgery.

**Table 3:** The predictive values of cytology and combined cyto-cell block results

	Cytology	Cyto-cell block
Sensitivity	93%	100%
Specificity	89%	90%
PPV*	88%	88%
NPV**	94%	100%
Accuracy	91%	94%

* PPV: positive predictive value; ** NPV: negative predictive value.

When these parameters were calculated considering the combined use of cyto-cell block the results were as follows: the sensitivity and specificity calculated were 100% (16/16) and 90% (20/22), respectively. The analysis of positive predictive value and negative predictive value revealed, respectively, 88% and 100%. The accuracy was 94% (36/38). The results included 2 false-positives for malignancy, 0 false-negative, 16 truly positive and 20 truly negative cases. Cases excluded from the analysis of sensitivity and specificity were as follows: 3 unsatisfactory cases (class I), 15 indeterminate cases (class III), 16 follicular lesion (class IV) and 3 PTMCs (Table 3).

## DISCUSSION

Here we further demonstrated the importance of US-FNA in the management of thyroid nodules. Additionally, we show that using cell block as a complementary diagnostic tool improves the efficiency of this technique. The overall accuracy of cyto-cell block for detection of neoplasm was 94%, with a sensibility and specificity of 100% and 90%, respectively.

Thyroid nodules are a significant clinical problem in the general population but the majority of them are benign and do not require surgery (15). Indeed, the prevalence of thyroid carcinomas is approximately 4%. The main gains from implementation of US-FNA include a reduction in the number of outpatient clinic attendances and hospital inpatient stays, with a consequent reduction in leave of absence from work. A faster diagnosis should also reduce patient anxiety (16). A reduction in operative rates for thyroid nodules would also produce a lower population rate of thyroid surgery-related morbidity (17). In agreement with the literature, we found that 6% of cases that were malignant or suspicious for malignancy (5,18).

One of the limitations of cytological analysis obtained by US-FNA is the relative high rate of unsatisfactory samples (5-30%), due to limited material available for adjunctive diagnostic investigations. Cellularity in cytological samples is an item to be considered, because often the limited representativeness of the sample determines the failure diagnosis. The difficulties to obtain satisfactory samples are inherent to the procedure and are consistent with the literature. Very fibrous nodules, cysts or necrotic lesions generally provide a very small number of viable cells for diagnosis (5,9,19).

In this context, the cell block technique allows recovery of small tissue fragments in a fluid sample which is processed to form a paraffin block. It has been widely accepted that this method of analysis increases the cell yield, providing a larger amount of material for analysis and improves diagnostic accuracy (12,20,21). However, the success of the cell block technique involves care and must meet certain requirements: maintenance of cell morphology and tissue architecture; optimization of the processing time without loss of quality material and obtaining sufficient reserve of material for further testing and immunohistochemical reactions (10). In this study, comparing the unsatisfactory rates, we can observe that the addition of the cell block to the US-FNA cytological technique significantly reduced the rate of unsatisfactory samples (17.1% vs. 4.3%, P < 0.001).

The embedded cytological sample in paraffin, with further processing, like a histological sample mimics the cell arrangement and the architectural features in the tissue, thus contributing to the diagnosis. Thus, it is disappointing that we find only few reports in the literature on the application of the cell block method to hospital routines. Our results show that the combination of the cell block and US-FNA techniques improved representativeness of the samples and a gain in diagnosis, since this methodology contributed to the increased cellularity and morphological details as groups with papillary and three-dimensional arrangement, cytoplasmic and nuclear preservation (22-24).

The number of insufficient/not diagnostic cases reached 4% in our series and indeterminate cytology was around 11%. These low percentages emphasize the US role in reducing the percentage of insufficient material or inappropriate diagnosis. These good results are due mainly to the guided choice of the nodule region to be aspirated, especially in the case of mixed nodules. The validated sensitivity (100%) and specificity (90%) of the diagnostic method were considered adequate. The accuracy (94%) of the procedure was considered high.

The major contribution of cell block analysis to FNA cytology in the present study was to decrease the rate of unsatisfactory specimen. Likewise, only a benign case in cytology change to malignant category after analysis of the cell block. Therefore, cell block preparation may change patient management like avoiding repeated FNA or unnecessary surgeries, once most of the benefit came from moving cases from the unsatisfactory category to the benign group.

In conclusion, US-FNA is a powerful diagnostic tool for the diagnosis of thyroid nodules and can effectively reduce unnecessary surgeries. The combination of techniques, cytology and cell block, has increased the representativeness of the samples and lower false negative rates. The main advantage of cell block technique is to concentrate the cells in a limited field, without loss of material for analysis and it can be used routinely. There is also the possibility of implementing special staining immunohistochemistry when needed.
